# Artifacts annotations in anesthesia blood pressure data by man and machine

**DOI:** 10.1007/s10877-020-00574-z

**Published:** 2020-08-12

**Authors:** Wietze Pasma, Esther M. Wesselink, Stef van Buuren, Jurgen C. de Graaff, Wilton A. van Klei

**Affiliations:** 1 Department of Anesthesiology, University Medical Center Utrecht, Utrecht University, Heidelberglaan, 100 3584XC Utrecht, The Netherlands; 2 Netherlands Organization for Applied Scientific Research and Department of Methodology & Statistics, Faculty of Social and Behavioural Sciences, University of Utrecht, Utrecht, The Netherlands; 3 Department of Anesthesiology, Erasmus MC – Sophia Children’s Hospital, Rotterdam, The Netherlands

**Keywords:** Artifacts, Anesthesia, Physiologic data, Machine learning

## Abstract

Physiologic data from anesthesia monitors are automatically captured. Yet erroneous data are stored in the process as well. While this is not interfering with clinical care, research can be affected. Researchers should find ways to remove artifacts. The aim of the present study was to compare different artifact annotation strategies, and to assess if a machine learning algorithm is able to accept or reject individual data points. Non-cardiac procedures requiring invasive blood pressure monitoring were eligible. Two trained research assistants observed procedures live for artifacts. The same procedures were also retrospectively annotated for artifacts by a different person. We compared the different ways of artifact identifications and modelled artifacts with three different learning algorithms (lasso restrictive logistic regression, neural network and support vector machine). In 88 surgical procedures including 5711 blood pressure data points, the live observed incidence of artifacts was 2.1% and the retrospective incidence was 2.2%. Comparing retrospective with live annotation revealed a sensitivity of 0.32 and specificity of 0.98. The performance of the learning algorithms which we applied ranged from poor (kappa 0.053) to moderate (kappa 0.651). Manual identification of artifacts yielded different incidences in different situations, which were not comparable. Artifact detection in physiologic data collected during anesthesia could be automated, but the performance of the learning algorithms in the present study remained moderate. Future research should focus on optimization and finding ways to apply them with minimal manual work. The present study underlines the importance of an explicit definition for artifacts in database research.

## Introduction

Physiological data captured by anesthesia monitors are used for medical record keeping during anesthesia. Such data are stored in Anesthesia Information Management System (AIMS) databases along with other anesthesia record keeping data. These rich databases are widely used for clinical research, since the data are obtained without much extra effort or altering the clinical workflow. However, automatically collected monitor data often include erroneous data that are not reviewed before they are stored, which may bias research results [1, 2]. In daily practice the anesthesiologist ignores artifacts based on other information available and therefore clinical anesthesia care is not affected. For example, artifacts in the ECG signal caused by detached electrodes (‘asystole’) can be ignored in the operating room based on other monitoring (normal invasive blood pressure signal). When these same data are used for research purposes, the context of the procedure is lost, and it is harder to distinguish which measurements are true and which are artifacts [3, 4].

Usually researchers come up with a definition of artifacts, and apply this definition to the data to correct errors. For example, values above and below a certain threshold are defined as artifacts and consequently removed from the data. Several other solutions for artifact removal are available [5] and effectiveness and accuracy of these methods will depend on the situation in which the data were collected. There is no consensus on which method for artifact removal should be used in AIMS database research. Thus far the removal of artefactual data live by hand (with presence in the operating room) seems to be the gold standard, to which other methods are compared [6–9]. Manual cleaning of artefactual data is not only a cumbersome and time consuming process, but could also depend on the situation in which the data were collected, similarly to automatic filtering methods. Factors that can affect artifact annotation are for example: the time when the data were annotated (during the anesthesia procedure or afterwards) the location (presence in the operating room or remote location) and who annotated the data ( i.e. an anesthesiologist, or a researcher). The pre-existing definition of artifacts in the rater’s mind or defined in a study protocol will influence which values will be marked as artifacts. Therefore manual annotations and models based on these manual annotations are difficult to generalize [7, 10].

The aim of the present study was to compare different artifact annotation strategies, and subsequently to assess if a learning algorithm would be able to simulate the decision to accept or reject individual vital sign data points. We used invasive blood pressure measurements as an example and hypothesized that regardless of different annotation methods, an algorithm could be trained to perform with comparable accuracy. Such algorithm learning strategy could standardize automated artifact removal to clean data for clinical research.

## Methods

### Live observation

Two research assistants (last year medical students) were trained by an anesthesiologist, to observe and annotate anesthesia procedures, during a time period of 11 weeks (between July 29th and October 11th) in the University Medical Center Utrecht. The local ethics committees approved the protocol and waived the need for informed consent (University Medical Center Utrecht Medical Research Ethics Committee, protocol no. 19–629). At the start of each workday, the assistant identified non-cardiac procedures in adults with planned invasive blood pressure monitoring. Each measurement session covered a part of the anesthesia procedure of at least one hour, to ensure that a mix of procedures was sampled. Procedures were preferably visited after induction or before end of surgery. The visiting order was not randomly determined, rather procedures were visited in sequence (i.e. when an observation was finished, the research assistant would identify the next eligible procedure to visit). The research assistant registered observations on a laptop that was not connected to the operating room equipment. We used Behavioral Observation Research Interactive Software (BORIS, https://www.boris.unito.it, Torino, Italy) software to record live observations during anesthesia. This software package allows for swift registration of observations, using a keystroke per type of observation, ensuring fine granularity in the data [11].

Invasive blood pressure was measured with an IntelliVue monitoring system (type MP70, X2 multimeasurement module; Philips, Germany). To mark the beginning of a registration, the arterial catheter was flushed, which generated a flush artefact in the waveform recordings, which was used later. Observers were instructed to document the start and end of any disturbance of the waveform signal, displayed on the patient monitor. At the same time, the reason for this artifact period was documented. When the observer was uncertain about the artifact events, he was permitted to discuss the artifact with the clinician responsible for the anesthetic procedure. The different artifact reasons for blood pressure were flush, blood sampling, sensor issues or movement of the patient, simultaneous non-invasive blood pressure measurement and height of the pressure sensor. The observer described this reason as a free text comment. When the observer was not able to categorize the reason of an artifact, this was later discussed with the research team and categorized.

The BORIS live registration software was not able not register the true observation time, based on the true time of the AIMS database (Anstat, version 2.1, 2019, Carepoint, Ede, The Netherlands). Therefore we looked up the flush event in the stored waveform data. With the registered flush time at the beginning of the observation and the actual flush time we synchronized the live observations with the data points in the AIMS database. Waveform data were analyzed using SignalBase, version 10.0.0 (legal copyright: UMC Utrecht MTKF, Utrecht, the Netherlands). SignalBase was developed to review and analyze raw waveform data as stored in the AIMS database.

### Data registration and retrospective annotation

In the institutional AIMS one data point for each measurement minute of invasive blood pressure is stored. This value is calculated by taking the median of the previous measurement minute (which encompasses 12 data points outputted by the patient monitor at 1/5 Hz). The resulting data points (1/60 Hz) were annotated retrospectively by one of the researchers (E.W.). This annotation was done using an interactive *R shiny* application designed for this study (https://github.com/wietze314/annotate-vital-signs), which displayed data points from the AIMS database. The invasive blood pressure measurements were annotated, with the complete health record available, as an additional reference, but blinded by the artifact identification done by the live observant. Therefore the observer was also aware of events and medication administrations during anesthesia. Besides artifact annotations, the application also collected meta-data such as the start and end time of the annotation process per observation.

### Data preparation and definition of artifacts

**Fig. 1:**
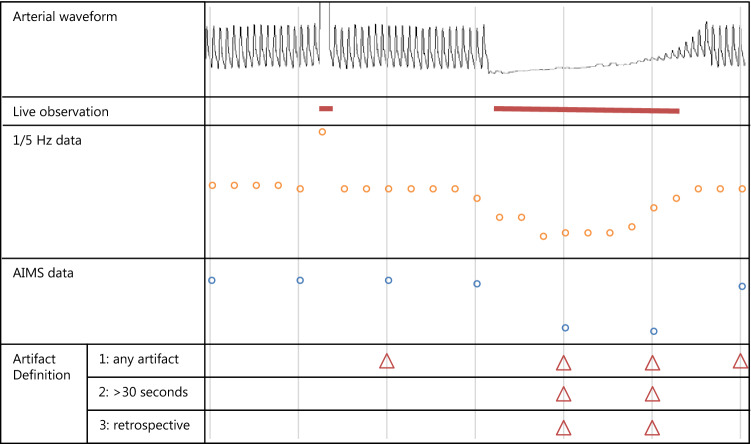
A simplified display of monitor and anesthesia information management system (AIMS) database data. Live artifact observations (red line) were based on the arterial waveform displayed on the patient monitor. Each minute (vertical lines) the AIMS software stored a data point (blue), which was based on the previous measurement minute (1/5 Hz data) (orange data points). The live observations were translated to artifacts by two definitions. Finally AIMS data points were retrospectively annotated. Definition 1: When there was any artifact during the measurement minute, the data point was identified as an artifact. Definition 2: When there was more than 30 s of artifact, during the measurement minute, the data point was identified as an artifact. Definition 3: Retrospectively identified artifacts, according to stored AIMS data points. Definition 4: The 1/5 Hz data points (orange) were considered artifacts, when they fell within a artifact period (red lines)

**Fig. 2:**
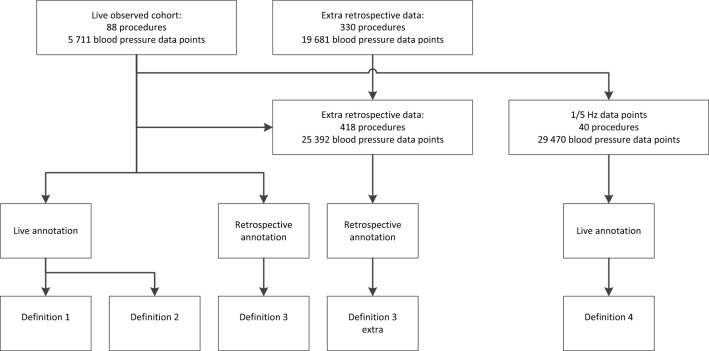
Flow diagram inclusion of observed procedures and which artifact definitions were applied. 88 procedures were live observed. 330 extra procedures were retrospectively collected for a post-hoc analysis

In order to compare the live observations to the retrospective annotations, we linked both datasets to the vital signs data in the AIMS database. During live observation the time period, in which the monitor displayed artifactual data, was registered. The data points stored in the AIMS database were based on a period of one minute of measurements. To combine these data, we calculated how much overlap each of the measurement minutes had with artifact periods. If there was any overlap (i.e. the AIMS data point was based on waveform data with artifacts), the data point was defined as an artifact (definition 1). Additionally, to generate a more specific artifact definition, we defined a data point as an artifact when there was more than 30 s of artifact during a measurement minute (definition 2). Furthermore, we marked individual data points as artifacts retrospectively, based on the available AIMS data (definition 3). The concept of data preparation is illustrated in Fig. 1. Apart from the data points stored each minute (1/60 Hz), we also collected 1/5 Hz data from a subsample of the cases, of which these higher resolution data were automatically stored in the AIMS database. We could not collect these data from all cases, because it required a change in settings of the AIMS software, which was set in only a part of the operating rooms for the purpose of this study. A 1/5 Hz data point was defined as an artifact, when this data point was within a live observed artifact period (definition 4) (Fig. 2).

In addition to the data collection of artifact periods, the observer also noted the cause of these artifactual periods. In the rare event of a data point which was influenced by multiple artifacts, the cause of artifact for that data point was determined by a majority vote, i.e. the cause that represented the most seconds of artifact was chosen. For example if within a measurement minute there was an artifact of 10 s because of blood sampling and subsequently an artifact of 30 s caused by manipulation of the blood pressure sensor, the data point was classified under the latter cause (Sensor issues or movement).

### Blood pressure processing

Our aim was to predict artifacts in AIMS data, using data normally available for retrospective database research. Therefore, we chose to only use vital signs data points (blood pressure and heart rate) to extract features for algorithm training. The feature types that we extracted and calculated were: systolic blood pressure, diastolic blood pressure, mean blood pressure, heart rate, pulse pressure (systolic–diastolic blood pressure), ratios between heartrate and blood pressure (systolic blood pressure divided by heartrate and diastolic blood pressure divided by heartrate), ratio between systolic and mean arterial blood pressure, and ratio between mean and diastolic arterial blood pressure. For each of the aforementioned features, the differences between the current data point and the five previous and five next data points were calculated. Thus in total eleven features were generated per feature type. Additionally, the median and the mean was calculated from the current data point and the five preceding and five following data points (11 data points), for heartrate, diastolic, systolic and mean blood pressure. Also the difference and the relative difference (difference divided by the median or mean) between the current data point and the median and mean was calculated. This process resulted in 111 features to present to a learning algorithm.

The same procedure was used for 1/5 Hz vital signs collected from the AIMS database, in a subset of the cohort. Only now the 15 preceding and 15 following data points for each data points were used, with an interval of 20 s between data points (i.e. skipping 3 measurements each time). This in turn, generated 291 features in total.

### Statistical analysis

Incidences of artifacts in different artifact annotation definitions were calculated, as well as differences between both observers. We then compared live and retrospective observations by creating contingency tables and calculating the sensitivity, specificity and positive predictive value. In this, the live artifact annotations (definition 1 and 2) were used as the reference. Finally we calculated the time which was used to annotate the data for the different artifact definition.

### Learning algorithms

For training learning algorithms, we considered each AIMS data point as an independent observation. The features were calculated using the surrounding data of each data point, thus providing the algorithms also with information of changes in time. All data points were first randomly assigned to the training and test set, with probability of 0.8 and 0.2 respectively. We generated a different training and test set for each learning algorithm method and each artifact definition, using different random seeds.

We used three different (machine) learning algorithms to model artifacts in invasive blood pressure data, i.e. lasso penalized logistic regression, a single layer neural network and a support vector machine [12]. First, we optimized the chosen learning algorithm. The training set was used to train the algorithm, and to tune the hyper parameters, with a fourfold cross-validation. The optimal performing set of hyper parameters was chosen based on the Kappa statistic. We chose Kappa as a performance measure over accuracy, because the incidence of the outcome (artifact) was rare. Kappa corrects for agreement by chance, and is more informative for highly skewed data [13]. We trained and optimized all four artifact definitions separately, thus each optimal model had dedicated hyper parameters. All algorithms were trained using the caret package in R [14]. We expressed performance of the different algorithms in sensitivity and specificity and positive predictive value, based on the reference artifact data, which we manually collected. Finally, we evaluated performance of the learning algorithm on the test set, which was kept separated from the algorithm learning procedure and cross-validation.

The *glmnet* method was used to train a lasso restrictive logistic regression [15]. The hyper parameter lambda was optimized, which defines how much the estimates are penalized, and therefor determines the generalizability of the model. Alpha was kept constant at one, which means that lasso regression is performed. A neural network was trained using the *nnet* method [16]. The neural network consisted of one hidden layer, which size we optimized with cross-validation, testing 2 to 20 units. In addition the hyper parameter weight decay was optimized setting its value from 10^–7^ to 10. The weight decay parameter determines how much estimates are penalized, and therefor determine how generalizable a model will be. Finally, we trained a support vector machine with a radial basis function or Gaussian kernel [17], using the *svmRadial* (*e1071* R package) method. The hyper parameter C and sigma were optimized with cross-validation. The C parameter defines how much estimates are penalized, and therefore determines the generalizability of the model, where a low C means more generalizable. C was varied from 5*10^–4^ to 10^3^. The sigma parameter (or gamma parameter) determines the reach of each data point, which influences which observations determine the decision boundary of the support vector machine. Low sigma will result in a more linear decision boundary and a lower variance model than when a higher sigma is used. We varied sigma from 5 × 10^–4^ to 0.2.

As a post-hoc analysis, we collected extra procedures which we annotated retrospectively, to see if the performance of learning algorithms might improve. These cases were randomly selected from January 1st to June 1st 2019. We only considered non-cardiac and non-thoracic surgery in adults. When there were issues with documentation (i.e. the health record was incomplete) the procedure was also excluded. In each selected procedure, a period of 60 min was randomly chosen for annotation. The middle of the procedure was preferred (higher probability of sampling), which was similar to the sampling strategy we used in the prospective cohort.

De-identified data collection and statistical analysis was performed with R (R Foundation for Statistical Computing, Vienna, Austria. https://www.R-project.org, R version 3.5.1 (2018–07-02)).

## Results

### Cohort

In total we included 88 procedures, which summed up to a total of 5711 blood pressure data points. Additionally, 29,476 blood pressure data points from 40 procedures captured at a higher frequency (1/5 Hz) were included (Fig. 2). Baseline characteristics for the observations are listed in Table 1.

**Table 1:** Baseline characteristics of observed procedures

Parameter	N (%) or Median (IQR)		
	All	Observer 1	Observer 2
Number of procedures	88	37 ( 100%)	52 ( 100%)
Age (years)	66 ( 56–74)	69 ( 57–76)	66 ( 55.5–74)
Durations of surgery (minutes)	344 ( 241–463)	327 ( 239–471)	356 ( 249–461)
Male	39 (44%)	19 ( 51%)	20 ( 38%)
Weight (kg)	76 ( 68.6–86.7)	76 ( 69.3–86.7)	75 ( 67.4–85.5)
Surgical specialty			
ENT and Maxillofacial	15 (17%)	8 (22%)	7 (13%)
General	30 (34%)	11 (31%)	19 (37%)
Neurosurgery	23 (26%)	10 (28%)	13 (25%)
Gynaecology and Urology	17 (19%)	5 (14%)	12 (23%)
Other	3 (3%)	2 (6%)	1 (2%)
ASA classification			
1	5 (6%)	1 (3%)	4 (8%)
2	47 (53%)	17 (47%)	30 (58%)
3 or 4	36 (41%)	18 (50%)	18 (35%)

Reported values are number of procedures (percentage) or median and interquartile range (IQR)

*ASA* American Society of Anesthesiologists, *ENT* ear nose and throat

### Artifacts

In 5711 blood pressure data points, 349 (6.1%) were based on data with an artifact, annotated by the observer (definition 1). According to definition 2 (at least 30 s of artifact), only 118 (2.1%) data points were identified as artifacts. Artifact incidence was 2.4% and 1.8% for observer 1 and observer 2, respectively. Retrospective artifact annotation (definition 3) yielded 124 (2.2%) artifacts (Table 2). Within the 40 cases that contributed 1/5 Hz data, we identified 761 (2.6%) of 29,476 data points as artifacts. For the post-hoc analysis we collected an additional 330 retrospective observations, with 19,681 blood pressure data points, of which 226 (1.1%) were identified as artifacts. The median time spent for retrospective annotation of 418 observations was 25 s (IQR 13–47 s)).

**Table 2:** Artifact incidence according to four artifact definitions

	N	(%)
Cohort blood pressure data points	5711	
Definition 1: Any artifact	349	(6.1)
Definition 2: > 30 s artifact	118	(2.1)
Definition 3: retrospective annotation	124	(2.2)
1/5 Hz blood pressure data points	29,470	
Definition 4: within artifact	761	(2.6)
Post-hoc additional data points	19,681	
Definition 3: retrospective annotation	226	(1.1)

Taking live annotation (definition 1) as the reference, retrospective annotation had a sensitivity of 0.14 and a specificity of 0.99, with a positive predictive value of 0.40. Comparing the more specific artifact definition 2 (at least 30 s of artifact) yielded a sensitivity of 0.32 and specificity 0.98, with a positive predictive value of 0.31. Table 3 lists both contingency tables and test parameters.

**Table 3:** Comparison of artifact annotations

Definition 3: Retrospective	Definition 1: any artifact		Definition 2: > 30 s of artifact	
	Artifact	No artifact	Artifact	No artifact
Artifact	49	75	38	86
No artifact	298	5289	80	5507
Sensitivity	0.14		0.32	
Specificity	0.99		0.98	
Positive predictive value	0.40		0.31	

Contingency tables for live observed artifacts (Definition 1 and 2), compared to retrospective annotation (Definition 3)

The most frequently reported cause of an artifact was sensor issues and/or movement of the patient (Table 4).

**Table 4:** Artifact causes

Cause of artifact	Definition			
	Definition 1: any artifact		Definition 2: > 30 s of artifact	
	Artifacts	Retrospective	Artifacts	Retrospective
Blood sampling	40(12%)	8(20%)	23(19%)	5(22%)
Sensor issues or movement	136(39%)	3(2%)	23(19%)	3(13%)
Flush	9(3%)	1(11%)	1(1%)	1(100%)
Height of pressure sensor	56(16%)	23(41%)	37(31%)	20(54%)
Simultaneous NIBP	68(19%)	4(6%)	18(15%)	3(17%)
Other/Not specified	38(11%)	10(26%)	16(13%)	6(38%)

*NIBP* non-invasive blood pressure

Artifact causes and retrospective identification

Results are presented as N (%)

Each second column’s percentage is calculated by dividing the retrospectively detected (true positives) by the total number of artifacts in that category

The data from the 418 procedures including the 88 live observed procedures, were published as part of this publication on dataverse.nl (https://doi.org/10.34894/3UNUTS).

### Learning algorithms

For each of the four artifact definitions, three different machine learning algorithms were fitted. Performance ranged from poor (definition 1, lasso regularized logistic regression, kappa 0.053) to moderate (definition 3: retrospective annotation, neural network, kappa 0.588). Few normal data points were marked as artifacts (false positives), therefore the learning algorithms overall had a high specificity. When the amount of data presented to the learning algorithm, increased (definition 3 with additional data) the performance also increased. For example, for support vector machine, kappa increased from 0.524 to 0.651 (Table 5).

**Table 5:** Learning algorithms, to predict artifacts in AIMS vital signs data

Artifact definition	Function	Hyper parameters	fourfold cross-validation				Performance test dataset			
			Kappa	Sens	Spec	PPV	Kappa	Sens	Spec	PPV
1: Any artifact	glmnet	alpha = 1, lambda = 2.78e-06	0.168	0.124	0.989	0.415	0.053	0.041	0.991	0.250
	nnet	size = 20, decay = 1e-07	0.166	0.240	0.939	0.209	0.087	0.158	0.941	0.123
	svmRadial	sigma = 0.001, C = 1000	0.216	0.204	0.974	0.329	0.201	0.200	0.969	0.304
2: > 30 s of artifact	glmnet	alpha = 1, lambda = 3.59e-05	0.285	0.241	0.992	0.401	0.066	0.062	0.992	0.100
	nnet	size = 8, decay = 0.01	0.226	0.277	0.979	0.221	0.118	0.154	0.976	0.129
	svmRadial	sigma = 5e-04, C = 1000	0.215	0.182	0.991	0.309	0.183	0.176	0.991	0.214
3: Retrospective annotation	glmnet	alpha = 1, lambda = 2.78e-06	0.389	0.339	0.995	0.671	0.447	0.407	0.991	0.524
	nnet	size = 20, decay = 0.001	0.426	0.353	0.994	0.597	0.588	0.500	0.996	0.733
	svmRadial	sigma = 5e-04, C = 100	0.530	0.438	0.996	0.716	0.524	0.429	0.995	0.706
3: Retrospective (additional data points)	glmnet	alpha = 1, lambda = 4.64e-04	0.462	0.315	1.000	0.923	0.399	0.275	0.999	0.759
	nnet	size = 10, decay = 1	0.560	0.469	0.997	0.721	0.481	0.386	0.997	0.659
	svmRadial	sigma = 5e-04, C = 50	0.552	0.431	0.998	0.790	0.651	0.521	0.999	0.884
4: 1/5 Hz data	glmnet	alpha = 1, lambda = 2.78e-06	0.245	0.169	0.997	0.592	0.100	0.056	0.999	0.615
	nnet	size = 9, decay = 1	0.468	0.395	0.993	0.611	0.538	0.486	0.993	0.627
	svmRadial	sigma = 0.005, C = 50	0.616	0.497	0.998	0.840	0.631	0.518	0.997	0.830

## Discussion

### Main findings

We compared different artifact annotation strategies of captured blood pressure data points in an AIMS database. Live annotated artifacts were frequently not identified as artifacts retrospectively (sensitivity of 0.32). The learning algorithms we subsequently developed to artificially identify artifacts were not able to adequately model artifacts which were annotated during live observations. Although the performance of these algorithms increased when retrospective annotations were modelled, the overall performance remained moderate.

Artifacts in invasive blood pressure measurements have different causes, such as movement or measurement technique artifacts. Some of the artifacts were short lasting and harder to pick up retrospectively, while others were longer lasting (Table 4). For example, movement artifacts according to artifact definition 1 were in only 2% of the cases identified as an artifact retrospectively (definition 3). On the other hand, the artifacts according to definition 2 (> 30 s of artefactual signal) were retrospectively identified in 13% of the cases. In the present study, the AIMS used a calculated median of one minute of data to store data points. It therefore makes sense that short lasting artifacts have not resulted in artifactual data points within the AIMS database, which could be identified retrospectively. Nonetheless, we would have expected a larger difference as a result of the effect described above. In addition, we found variation over different causes of artifacts in retrospective positively identified artifacts. These differences likely were a result of differences in information availability per situation. For example, from the AIMS record data points with systematic errors in blood pressure measurement due to the height of artery sensor placement were easier identified, than artifacts caused by movement.

In the present study we present different methods to manually define artifacts in AIMS data, and compare these different definitions with each other. Others have analyzed differences between artifact annotations, but comparisons were done to compare different raters, who received the same annotation task, i.e. retrospective annotation. The present study shows that it is not only important to describe who annotated data, but also when and how data points were marked as artifacts, in order to make research reproducible [7].

We have prospectively collected data during a period of twelve weeks. This resulted in a reasonable quantity of observations. Nevertheless, the incidence of artifacts in the present study was quite low (2%). The amount of data available for the learning algorithms might thus have been too small. We observed procedures mainly in the maintenance phase of surgery, as we expected that it would be more complex to label artifacts precisely in the induction and emergence period where a lot of things happen at the same time. The artifact incidence was similar to what was previously found during maintenance in pediatric surgery, which was also lower than during induction or emergence [3]. Furthermore the type of surgery could have affected the incidence of artifacts, for example the cohort had a high portion of neurosurgery procedures, during which movement of the patient is limited and the surgical field is further away from the blood pressure sensor than other types of surgery.

In the present study, only one researcher annotated the data retrospectively, which can be considered a limitation. We could have improved quality of annotation when more than one researcher had annotated the data. On the other hand, because differences between these raters also need to be evaluated, the time invested in an extra person who annotated the data, would have been considerably more than twice the time which we spent thus far. Also the goal of this research was not to compare raters with each other, as has already been done previously [7].

We used two definitions to translate the live observations to an artifact definition (Definition 1 and 2), using the duration of the artifacts. Another approach could have been to combine the severity of the artifact, for example the deviance from baseline, with the duration of the artifact. In theory short extreme artifacts (e.g. flush events) can affect analysis differently than long but less extreme artifacts (e.g. height of the pressure sensor). In our situation the duration of artifacts was more important since our system stores the median of 12 consecutive blood pressure measurements. Therefore we only used the duration of artifacts, but in other situations this definition might be too limited.

We used two research assistants for live observations, which could result in differences in the way data were annotated. We observed minor differences in artifact incidence in each group of procedures, which were probably due to differences in procedure types (Table 1). The number of artifacts according to retrospective annotation (definition 3) varied in a similar way, between these two subgroups (data not presented). Unfortunately, we have not performed a double-code observation to compare both observers adequately.

We have purposefully used only automatically collected physiologic data captured during the anesthetic procedure as source of features. We made this choice to ensure that resulting methodology and workflow will be generalizable, even when no other data than vital signs are available. This approach makes the methodology broadly applicable. On the other hand we tried to model a (human) decision, i.e. manual artifact identification, with limited information, from which the performance of the learning algorithms would have suffered. We saw that none of the learning algorithms performed well enough to apply for future research, as presented here. We showed in a post-hoc analysis that the performance could improve by adding additional data points. Nevertheless the information that was available to these algorithms was probably still too limited. To understand this concept better, future research could focus on adding not only more observations but also more features to the model, which are commonly available in databases used for research. For example patient characteristics, procedure type and medication administration or other events around the data point of interest could be added.

### Implications

Before we can say anything about the implications of this study we first need to consider the definition of an artifact. Is every measurement in an AIMS database, based on a disturbed or a not perfect signal an artifact? Or does a live observed disturbance in a signal only produce an artifact, when the stored data point is different than what we expect for a patient at that particular time during anesthesia? But in the latter case, how do we define an expected value? These questions show that artifact annotation is a subjective matter, and a question of definition. It is important that researchers report what they considered to be artifacts, even when this process was done manually.

Despite this issue in defining artifacts, artifact annotation could still be automated using learning algorithms. The present study showed that this is not straightforward and might still require an investment of time to collect manually annotated data points for training. We live observed around 95 h of anesthesia, while using retrospective data from 328 h only improved the performance of the learning algorithms marginally. Observing this much data live would have been very labor intensive and likely not feasible. In contrast, retrospectively annotating these data took us around three hours with a custom made registration application by a single person. This makes retrospective annotation better suitable to remove artifacts from research data, than live annotation.

Even though machine learning algorithms performed poorly in the present study, our approach is still insightful for those who want to apply similar annotation tools and models on their own AIMS data. Future research could focus on improving the performance and develop application of the methods presented in the present paper. To minimize time spend on manual data collection, we suggest optimizing this process using an active learning strategy. With this strategy, only data points are annotated, which contribute significantly to the learning algorithm. This could reduce the time spend on annotating data significantly [18, 19].

## Conclusion

Identification of artifacts in invasive blood pressure measurements depends on the moment of annotations (live versus retrospective) and the person who annotated the data points. Nevertheless, these different artifact definitions could be modelled with learning algorithms in a similar way. The performance of these algorithms was poor in the present study and should be improved before applying in the future. A substantial amount of manually annotated data is still required to train these algorithms. As a positive by product of such an effort, researchers are forced to define explicitly what artifacts in their data are.
